# Thrombus or tumor? a case of fibroelastoma  as indicated during the submission process

**DOI:** 10.1186/1757-1626-2-31

**Published:** 2009-01-08

**Authors:** Enrico Vizzardi, Pompilio Faggiano, Elena Antonioli, Gregoriana Zanini, Ermanna Chiari, Savina Nodari, Livio Dei Cas

**Affiliations:** 1 Section of Cardiovascular Disease, Department of Applied Experimental Medicine, Brescia University, Brescia, Italy

## Abstract

We describe the case of a 50-year-old woman who was admitted to a pheriferal department for heart failure. The echocardiography revealed a small mass measuring about 1.3 × 1.0 cm adhering to the non-coronary cusp of the aortic valve, mild dilated cardiomiopathy and severe biventricular dysfunction. This mass had erroneously been considered a thrombotic lesion, so the patient was treated with thrombolysis and heparin e.v. Only after a transoesophageal echocardiography a tumour cardiac mass was suspected. The diagnosis of fibroelastoma was confirmed by MRI and then from the anatomic and histoligical definition after surgery.

## Introduction

Cardiac papillary fibroelastoma (PFE) is a rare primary cardiac neoplasm of unknown prevalence [1]. Since the introduction of echocardiography, the diagnosis of these tumours in living patients has been reported sporadically. The largest report of pathologically confirmed PFE includes only 17 patients [2]. Echocardiographically, this entity has been described as a small, well-delineated, pedunculated mass with a predilection for valvular endocardium [1-3]. Case reports have associated PFE with coronary, cerebral, pulmonary and retinal artery emboli, although the frequency with which this occurs is not well established [4,5].

## Case presentation

We describe the case of a 50-year-old woman who was admitted to a pheriferal department for heart failure (NHYA class III) in mild idiopathic dilated cardiomyopathy. She had chronic atrial fibrillation and hypertension in anamnesis. On admission to the hospital, the patient's blood pressure and heart rate were normal. Her oral temperature was 36.5°C. Examination of the head and neck revealed no abnormalities; the external jugular vein was distended; no adenopathy was present. Abdominal examination revealed mild hepatomegaly. Cardiac examination revealed a systolic murmur at the fourth intercostal space on the marginal sternal line. The laboratory results were normal; no increase in inflammatory index. Chest radiograph showed an enlarged cardiac silhouette and the ECG showed atrial fibrillation. The patient also underwent echocardiography, which revealed a small mass measuring about 1.3 × 1.0 cm adhering to the non-coronary cusp of the aortic valve, mild dilated cardiomiopathy and severe biventricular dysfunction. In order to the presence of AF, this mass had erroneously been considered a thrombotic lesion, so the patient was treated with thrombolysis and heparin e.v.

**Figure 1:**
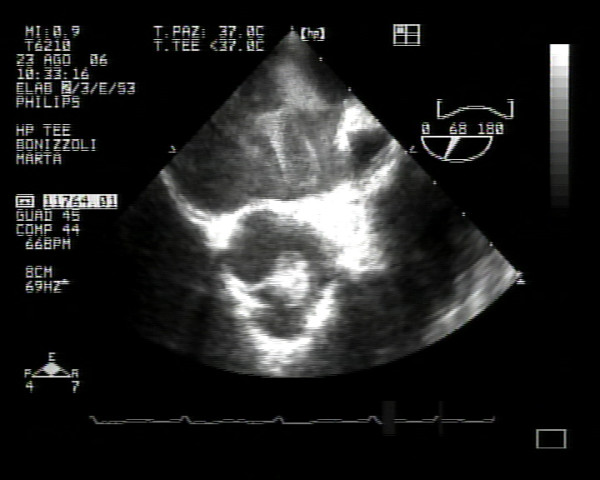
**Evidence of a mass on the noncoronary aortic cusp**.

**Figure 2:**
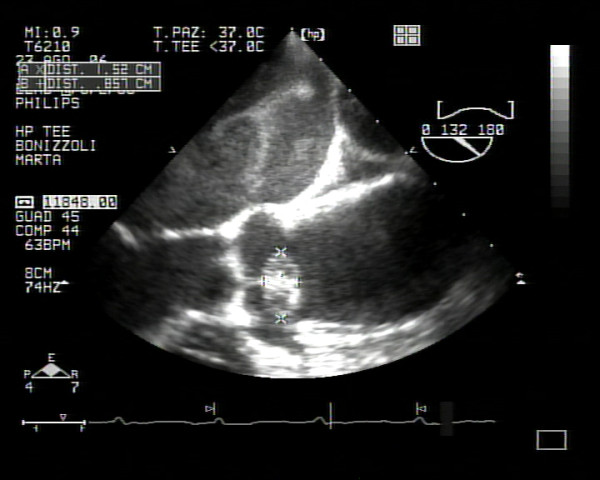
**Evidence of a mass on the noncoronary aortic cusp**.

After fibrinolysis, the dimension of this lesion did not change, so the patient was transferred to our department. Transthoracic and transoesophageal echocardiography was performed to define the mass. It showed a highly mobile, homogeneous echodense mass with a smooth surface from a pedicle to the closing margins of the noncoronary aortic cusp. All three aortic cusps had no signs of thickening and appeared normal as judged by echocardiography. Colour Doppler revealed no valvular incompetence. From echocardiographic findings, a tumour of the aortic valve rather than a thrombus or a vegetation was suspected (Figs. 1 and 2). Magnetic resonance imaging (MRI) was then performed to determine the character of the tumour tissue. It revealed a mobile nodular mass which was characterized by a slightly lower signal intensity than the myocardium at MRI with a single-slice breath-hold segmented turbo fast low-angle shot technique.

To prevent systemic embolization, the patient underwent excision of the tumor. The echocardiographic and MRI findings were confirmed during surgery. A 1-cm papillary mass was found attached to the noncoronary cusp of the aortic valve and was treated by simple excision of the tumour without aortic valve replacement. Histological examination revealed several fragments covered by flattened cells. The core was composed of dense fibrous tissue with hyalinization, consistent with a papillary fibroelastoma of the endocardium.

In the following days, the patient no longer showed signs of dyspnoea. An echocardiogram was performed at discharge without evidence of a mass attached to the noncoronary aortic cusp.

## Discussion

Papillary fibroelastoma (PFE) is a benign, rare, gelatinous tumour derived from the endocardium; concerning frequency, it corresponds to the third most common primary intracardiac tumour, preceded by myxomas and lipomas [6]. Approximately 90% of PFE affects cardiac valves, usually as a single lesion, on the atrial face of atrioventricular valves or on any of the sides of semilunar valves. They rarely occur as single lesions.

Approximately 44% of PFE are found in aortic valves, followed by the undertaking of mitral valve in 35% of cases, tricuspid valves in 15%, and pulmonary valves in 8% [7]. Reports of cases of those tumors have demonstrated undertaking of all endocardial surfaces, including papillary muscles, tendinous chords, the septum or free walls of any of the cardiac chambers [8].

The size of described PFE varies from 0.1 to 4 cm, and most of them are smaller than 1 cm in diameter [9]. The age of the patients is variable. Most cases are described in adults over the age of 50, and there is no difference between the sexes [7,9,10].

PFE is an incidental finding in most cases, although among symptomatic patients the clinical presentation is variable and dependent on location, motility and size of tumour [7-9]. As most of it originates from left chambers, the most feared complication is systemic embolization, especially in cerebral or coronary circulation [7-9]. The most common clinical presentation described is stroke or transitory ischemic attack. Other described manifestations are: angina, myocardial infarction, sudden death, heart failure, syncope, pulmonary embolism, blindness, peripheral embolism with renal infarction [7]. In aortic valve tumour patients, sudden death and myocardial infarction are the most common manifestations.

Electrocardiographic findings are non-specific. Thoracic radiography may demonstrate signs of increase of cardiac chambers, pulmonary hypertension or congestion, especially if the tumour is occluding the mitral valve. Transthoracic, particularly transoesophageal, echocardiograms are the ideal method for tumour diagnosis and characterization, as they usually show the mass with its varied proportions, motile or not, well-delimited, pedunculated or sessile, of round, oval or irregular shape. They are mostly small (99% less than than 2.0 cm). Magnetic resonance imaging reveals the mass in valve leaflet or cardiac chamber, and the presence of enhancement with gadolinium in the tumoral mass increases the suspicion level [7]. Cardiac catheterization does not contribute to the diagnosis.

PFE has a characteristic appearance, resembling a sea anemone, with multiple ramifications held by a pedicle to the endocardium. At histological examination, it consists of an endothelium coat, which covers a connective tissue matrix with variable amounts of collagen, smooth muscle cells and elastic fibres [9].

For symptomatic patients, surgical exeresis is the choce of treatment, in an attempt to preserve valve tissue and function [11]. Among asymptomatic individuals, surgical procedure is controversial, as tumoral motility is the determining factor for surgical indication, for being ? an independent embolization and death predictor. Surgery is curative and there is no report of recurrence [7-9]. Most investigators recommend an aggressive approach to PFE, especially for lesions of the left side of the heart. Surgical excision of the PFE is considered to be curative and, in most cases, the tumour can easily be removed because of its pedunculation. In the series of Gowda et al. [7], a valve-sparing procedure was performed in 90% of patients referred for surgical excision of the cardiac PFE.

Management before a left side isolated lesion includes surgical removal, when the mass is big and/or movable or in the precence of patent oval foramen due to the possibility of paradoxical embolism [12]. When the patient is not considered for surgical removal of the PFE, long-term oral anti-coagulation is recommended, although no randomized controlled data are available.

## Conclusion

Transoesophageal echocardiography is proving to be superior to transthoracic echocardiography, not only in confirming the precence of a cardiac mass but also in defining its attachment to the underlying tissue, its exact location and its relation with surrounding structures [13].

In our case transthoracic echocardiography wasn't be able to correctly diagnosed fibroelastoma and so it managed to a wrong diagnosis and so to a wrong therapy. So there is the extreme necessity to perform always a transoesophageal echocardiography and particularly when a scarce acoustic window is present at the transthoracic one in order to give the correct diagnosis and the correct theraphy. In our case the probability that the mass could be a thrombus was very low. There are no definitive features on TTE that distinguish papillary fibroelastoma from other intracardiac masses. The sensitivity and specificity of TTE are in the order of 88.9% and 87.8%, respectively, with an overall accuracy of 88.4%, when the tumour is larger than 0.2 cm. When the tumour is less than 0.2 cm, the overall sensitivity of TTE is 61.9%, compared with 76.6% for TEE [12]. Single papillary fibroelastomas are detected by TTE in 91.4% of cases, whereas multiple fibroelastomas are only detected in 8.6%.

Echocardiographic diagnosis is not easy, particularly when PFE is small in size, when an associated lesion is masking the tumour, when there is a lack of significant characteristics to differentiate PFE from degenerative valve disease, and when the index of suspicion during the examination is insufficient [12]. Differential diagnoses include thrombi, infective or noninfective vegetations, degenerative valve tissue and other cardiac tumours: clinical data, blood cultures and laboratory tests, together with echocardiography [12].

However, it is almost impossible to define tissue character using this tecnique alone, as in our case. Magnetic resonance imaging excels at making a differential diagnosis of various soft tissues and organs because of its high resolving power [14]. Cardiac MRI gives superior visualization of cardiac tumours, and of metastasis if present [15].

We have presented a case of fibroelastoma that was initially incorrectly diagnosed by transthoracic echocardiography; it was detected and diagnosed by TTE echocardiography and magnetic resonance imaging. In cases of cardiac and valvular tumour, transoesophageal echocardiography and magnetic resonance imaging play important roles in making a diagnosis and selecting the optimal surgical treatment.

## Consent

Written informed consent was obtained from the patient for publication of this case report and accompanying images. A copy of the written consent is available for review by the Editor-in-Chief of this journal.

## Competing interests

The authors declare that they have no competing interests.

## Authors' contributions

EC and PF performed transthoracic and thransesophageal echocardiography. EV, GZ and EA treated and follow-up the patient during the admission and the stay in the hospital. SN and LDC gave us the supervision.
